# Impact of leaflet resection technique on mitral valve mobility after mitral valve repair assessed by echocardiography

**DOI:** 10.1186/s13019-025-03626-0

**Published:** 2025-10-21

**Authors:** Norihisa Yuge, Susumu Manabe, Daiki Hirayama, Ryuki Yamada, Mariko Hori, Koichiro Sugimura, Teruaki Ushijima, Hiroaki Shimokawa

**Affiliations:** 1 Department of Cardiovascular Surgery, International University of Health and Welfare Narita Hospital, 852 Hatakeda Narita, Chiba, 286-8520 Japan; 2 Department of Cardiology, International University of Health and Welfare Narita Hospital, Narita, Japan; 3 Department of Cardiovascular Surgery, International University of Health and Welfare Hospital, Nasushiobara, Japan; 4 Graduate School, International University of Health and Welfare, Chiba, Japan

**Keywords:** Mitral valve repair, Leaflet resection, Mitral leaflet mobility

## Abstract

**Background:**

Two surgical techniques, leaflet resection and leaflet preservation, are widely used for mitral valve repair in severe mitral regurgitation due to posterior mitral leaflet prolapse. However, which technique is superior remains unclear. The leaflet resection technique may affect the postoperative mitral valve function differently from the leaflet preservation technique because it causes anatomical alterations in the corrected leaflet. We aimed to evaluate the effect of the leaflet resection technique on mitral valve mobility, compared with that of the leaflet preservation technique.

**Methods:**

Forty-one patients underwent mitral valve repair for P2 prolapse. Among them, 27 underwent leaflet preservation and 14 underwent leaflet resection. We examined the effects of the leaflet resection technique on the mitral valve mobility.

**Results:**

Postoperatively, the mobility of the corrected leaflet was significantly decreased in the leaflet resection group (leaflet preservation: 35.1 ± 13.8 vs. leaflet resection: 22.7 ± 13.7°, p = 0.009). Particularly, the maximum closed angle was significantly decreased (leaflet preservation: 29.1 ± 11.4° vs. leaflet resection: 40.7 ± 12.0°, p = 0.004). Therefore, the closing motion of the resected leaflet was more restricted than its opening motion. Mitral valve function, including mitral valve area, and peak and mean transmitral pressure gradients were comparable in both groups.

**Conclusions:**

Although there were no differences in the mitral valve function, the leaflet preservation technique may be more effective than the leaflet resection technique at preserving the corrected leaflet mobility.

## Introduction

Mitral valve regurgitation (MR) is the most common valvular dysfunction, affecting 2% of the general population [1]. MR is a progressive disease, and the prevalence of severe MR increases with age [2]. Mitral valve (MV) prolapse is the most frequent cause of degenerative MR findings. MV repair is the generally accepted “gold standard” therapy for patients with severe MR due to MV prolapse, because of its excellent long-term results compared to MV replacement [3, 4]. For several years, resection of the prolapsing segment, including quadrangular resection with or without sliding plasty, and triangular resection has been the standard surgical technique for prolapse of the posterior mitral leaflet [5, 6]. In addition to these leaflet resection techniques, various artificial chordal replacement techniques (leaflet preservation techniques), using expanded polytetrafluoroethylene (e-PTFE) sutures, are widely available [7–9]. Both MV repair techniques are associated with excellent results in terms of low mortality, incidence of recurrent MR, and risk of reoperation [10]. The choice of surgical technique for MV repair is left to the discretion of the surgeon because it remains unclear whether leaflet resection or leaflet preservation is the best choice for MV repair due to posterior mitral leaflet prolapse. However, the leaflet resection technique causes morphological changes in the mitral valve leaflet, which may produce different effects on MV function compared with those of the leaflet preservation technique. In this study, we aimed to evaluate the effect of the leaflet resection technique on MV mobility compared with that of the leaflet preservation technique.

## Methods

### Design and patients

**Fig. 1:**
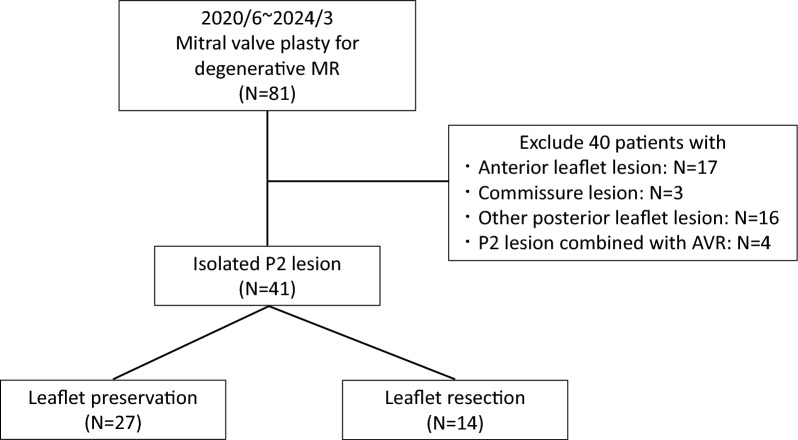
Flowchart of patient enrollment. MR, mitral regurgitation; AVR, aortic valve replacement

This study is a retrospective study. Between June 2020 and March 2024, 81 patients underwent MV repair for severe MR at two registered hospitals (Fig. 1). Among them, those who underwent mitral valve repair for anterior leaflet prolapse (N = 17), presented with commissure leaflet prolapse (N = 3), P1 or P3 lesions (N = 16), or P2 prolapse combined with aortic valve replacement (AVR) were excluded. Therefore, 41 patients who underwent MV repair for severe MR due to P2 prolapse were included in the present study. Of these patients, 27 underwent MV repair using the leaflet preservation technique and 14 underwent MV repair using the leaflet resection technique. The study protocol was approved by the Institutional Review Board of the International University of Health and Welfare Narita Hospital (22-Im-006).

### Operative technique

All MV repairs were performed via median sternotomy using cardiopulmonary bypass and cardioplegic arrest. The MV was exposed through a superior trans-septal approach or a right-sided left atriotomy approach. Mitral annuloplasty using a complete ring (Carpentier-Edwards Physio II ring; Edwards Lifesciences, Irivine, California, USA) or a partial ring (Physio Flex ring; Edwards Lifesciences, Irivine, California, USA) was routinely performed in all patients, and the choice of ring was left to surgeon’s discretion. The size of annuloplasty was determined intraoperatively based on the intertrigonal distance. In the leaflet preservation technique, the prolapsed leaflets were corrected by artificial chordal implantation without leaflet resection. Leaflet resection technique was planned to be used in patients with excessive or stiff leaflet tissues. However, no cases were found in which leaflet resection was necessary due to the presence of stiff leaflet lesions. Therefore, leaflet resection techniques were used in patients with excess leaflet tissue. All patients who underwent leaflet resection also underwent quadrangular resection with sliding plasty. The suture line of the resected leaflet was closed using either continuous or interrupted 5–0 prolene sutures. Artificial chordal implantation was performed using the tourniquet technique with CV-4 or CV5 ePTFE sutures (L. Gore & Associates, Inc., Flagstaff, Ariz) [9]. Even in the leaflet resection group, artificial chordal implantations for prolapsed leaflets were performed as required to obtain adequate coaptation. Concomitant MV repair techniques such as cleft closure technique were incorporated at the surgeon’s discretion. All patients with atrial fibrillation also underwent the maze procedure and left atrial appendage closure during the same operation. Surgery was performed at two registered hospitals by several surgeons experienced in the field of mitral valve plasty.

### Transthoracic echocardiography assessments

**Fig. 2:**
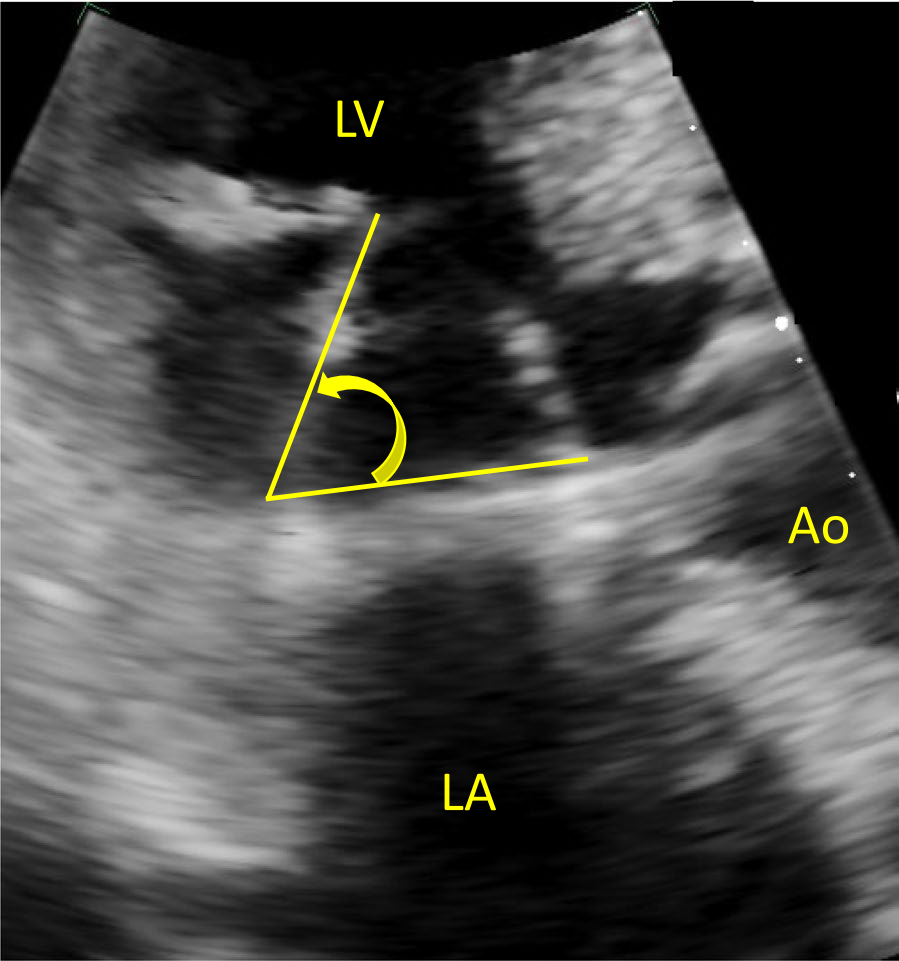
Measuring method of mitral leaflet mobility in the apical 3-chamber view. LA, left atrium; LV, left ventricle; Ao, Aorta

All echocardiographic assessments were performed at rest according to current guidelines [11, 12]. Mitral leaflet configuration was measured in the apical 3-chamber view (Fig. 2). To analyze the mitral leaflet configuration, the angles from the annular plane to the maximum open position and maximum closed position of both the anterior and posterior leaflets were measured. Left ventricular ejection fraction (LVEF) was calculated using the modified Simpson’s rule. Experienced sonographers with a deep understanding of echocardiography performed all examinations. When evaluating leaflet mobility and other clinical parameters, the sonographers performing the echocardiography was blinded to the procedure that the patient had undergone. Furthermore, we excluded all segment lesions other than P2 prolapse in order to accurately assess corrected leaflet mobility by eliminating measurement errors in echocardiography.

### Statistical analysis

Statistical analyses were performed using the Statistical Package for Social Sciences, version 23 (SPSS, Chicago, IL). Continuous variables are presented as mean ± standard deviation, whereas categorical variables are presented as frequencies and percentages of the total. Continuous variables were compared with a 2-sample t-test. Results were considered significant if *p* values were less than 0.05.

## Results

### Preoperative variables

The baseline characteristics and echocardiographic hemodynamic findings of the two study groups are summarized in Table 1. Patients in the leaflet resection group were significantly younger than those in the leaflet preservation group. However, sex distribution, body surface area (BSA), and the number of patients with preoperative atrial fibrillation were comparable between the two groups. Preoperative cardiac function parameters were comparable in both groups. The preoperative MV hemodynamic parameters were comparable between the two groups; however, mitral valve area (MVA) tended to be greater in the leaflet resection group (p = 0.13). MV mobility was similar in both groups in either the anterior or posterior leaflet (Table 2).

**Table 1:** Patients’ baseline characteristics

	Leaflet preservation (n = 27)	Leaflet resection (n = 14)	P value
**Patients’ characteristics**			
Age, yrs	69.3 ± 9.7	60.0 ± 13.2	0.01
Female sex, n (%)	8(29.6)	6(42.9)	0.41
BSA, m^2^	1.64 ± 0.22	1.64 ± 0.16	0.98
Atrial fibrillation, n (%)	10(37.0)	3 (21.4)	0.32
**Echocardiographic characteristics**			
LVEDV, ml/m^2^	123.8 ± 45.6	144.4 ± 54.2	0.21
LVESV, ml/m^2^	43.0 ± 19.6	55.3 ± 34.7	0.16
LVEF, %	65.8 ± 6.6	64.7 ± 8.8	0.64
LAVI, ml/m^2^	60.5 ± 22.2	67.8 ± 34.3	0.53
**Preoperative mitral valve characteristics**			
ERO, mm^2^	0.68 ± 0.33	0.67 ± 0.23	0.87
MVA, cm^2^	3.94 ± 1.07	4.60 ± 1.18	0.13
peak PG, mmHg	5.84 ± 4.02	3.80 ± 2.19	0.16
mean PG, mmHg	1.55 ± 1.30	1.00 ± 1.04	0.25

BSA, body surface area; LVEDV, left ventricle end-diastolic volume; LVESV, left ventricle end-systolic volume; LVEF, left ventricle ejection fraction; LAVI, left atrial volume index; ERO, effective regurgitant orifice area; MVA, mitral valve area; PG, pressure gradient

**Table 2:** Preoperative mitral valve morphological characteristics

	Anterior leaflet			Posterior leaflet		
	Leaflet preservation (n = 27)	Leaflet resection (n = 14)	P value	Leaflet preservation (n = 27)	Leaflet resection (n = 14)	P value
Maximum open angle, degree	72.2 ± 12.3	69.2 ± 7.3	0.44	67.6 ± 12.2	64.1 ± 19.5	0.50
Maximum closed angle, degree	18.5 ± 8.2	19.9 ± 12.0	0.67	−3.0 ± 16.2	−4.0 ± 17.4	0.87
Range of leaflet motion, degree	51.7 ± 19.1	49.3 ± 15.6	0.43	70.6 ± 21.3	68.1 ± 24.2	0.74

### Operative data

The intraoperative characteristics of the two groups are presented in Table 3. Complete rings were used significantly more frequently in the leaflet preservation group (leaflet preservation group: 92.6% vs. leaflet resection group: 57.1%, p < 0.01), whereas the partial rings were used more frequently in the leaflet resection group (leaflet preservation group: 7.4% vs. leaflet resection group: 42.9%, p < 0.01). The annuloplasty ring size and indexed ring size (implanted annuloplasty ring size/body surface area) were not significantly different between the two groups. The type of leaflet resection was quadrangular resection with sliding plasty in all patients in the leaflet resection group. The cleft closure technique was performed at similar frequencies in either group. There was a significant difference in concomitant tricuspid valve annuloplasty between the two groups (leaflet preservation group: 37.0% vs. leaflet resection group: 7.1%, p = 0.04). Maze procedure was performed at similar frequencies.

**Table 3:** Intraoperative characteristics

	Leaflet preservation (n = 27)	Leaflet resection (n = 14)	P value
**Type of annuloplasty ring**			
Complete ring, n (%)	25 (92.6)	8 (57.1)	< 0.01
Partial ring, n (%)	2 (7.4)	6 (42.9)	< 0.01
Annuloplasty ring size, mm	31.6 ± 2.4	31.9 ± 2.5	0.78
indexed ring size, mm/BSA	19.1 ± 2.5	19.4 ± 2.2	1.00
**Concomitant mitral valve repair technique, n (%)**			
Cleft closure	2 (7.4)	3 (21.4)	0.20
**Concomitant procedures, n (%)**			
TAP	10 (37.0)	1 (7.1)	0.04
Maze procedure	10 (37.0)	3 (21.4)	0.32

BSA, body surface area; CABG, coronary artery bypass grafting; TAP, tricuspid valve annuloplasty

### Postoperative outcomes

**Fig. 3:**
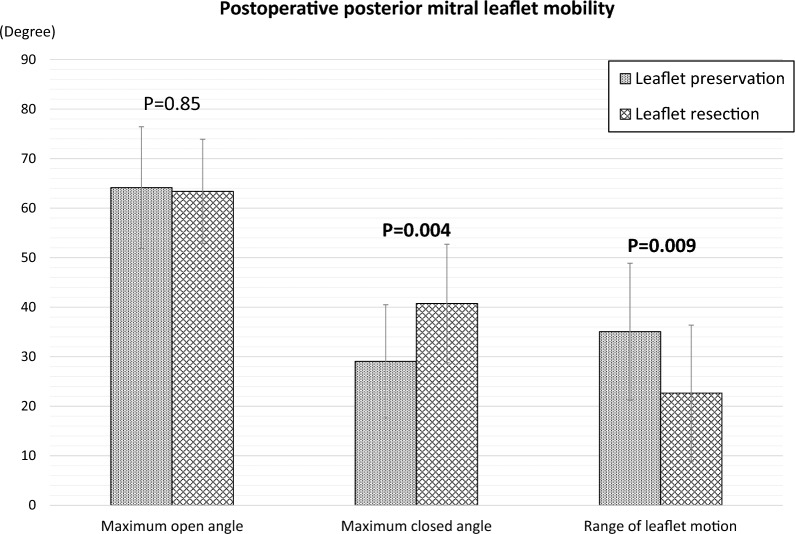
Comparison of postoperative mitral valve leaflet mobility

The patient’s postoperative findings are presented in Table 4. On average, a postoperative echocardiogram was obtained 9 days after surgery in all patients. Two patients (4.7%) were found to have residual mild MR in the leaflet preservation group; however, no moderate or severe MR was observed in either group. Left ventricular volumes were similar in both groups. LVEF tended to be lower in the leaflet resection group than in the leaflet preservation group (leaflet preservation group: 59.4 ± 8.6% vs. leaflet resection group: 53.9 ± 9.3%, p = 0.07). Regarding postoperative MV mobility, the maximum open angle of the corrected leaflet (leaflet preservation group: 64.1 ± 12.3° vs. leaflet resection group: 63.4 ± 10.5°, p = 0.85) did not differ significantly between the two groups. However, the maximum closed angle of the corrected leaflet was significantly higher in the leaflet resection group (leaflet preservation group: 29.1 ± 11.4° vs. leaflet resection group: 40.7 ± 12.0°, p = 0.004). Therefore, the mobility of the corrected leaflet was significantly decreased in the leaflet resection group (leaflet preservation group: 35.1 ± 13.8° vs. leaflet resection group: 22.7 ± 13.7°, p = 0.009) and restricted at the semi-opened position compared with that in the leaflet preservation group (Fig. 3). There was no significant difference in the MV parameters, including MVA, peak and mean pressure gradients, between the two groups.

**Table 4:** Postoperative characteristics

	Leaflet preservation (n = 27)	Leaflet resection (n = 14)	P value
**Postoperative echocardiographic characteristics**			
LVEDV, ml/m^2^	108.7 ± 27.6	111.4 ± 47.0	0.83
LVESV, ml/m^2^	45.2 ± 17.9	52.6 ± 28.1	0.31
LVEF, %	59.4 ± 8.6	53.9 ± 9.3	0.07
LAVI, ml/m^2^	42.8 ± 15.3	50.7 ± 18.6	0.16
**Postoperative mitral valve characteristics**			
MVA, cm2	2.67 ± 0.68	2.45 ± 0.54	0.28
Peak PG, mmHg	6.63 ± 2.66	6.89 ± 2.18	0.76
Mean PG, mmHg	2.19 ± 0.92	2.30 ± 0.59	0.68

LVEDV, left ventricular end-diastolic volume; LVESV, left ventricular end-systolic volume; LVEF, left ventricular ejection fraction; LAVI, left atrial volume index; MVA, mitral valve area; PG, pressure gradient

## Discussion

We evaluated the effect of the leaflet resection technique on the MV mobility, and compared it with that of the leaflet preservation technique for P2 prolapse. In this study, the leaflet resection group had significantly decreased corrected posterior leaflet mobility compared to the leaflet preservation group. In particular, the closing motion of the corrected leaflet during systole was markedly restricted. As a result, we showed that the corrected leaflet motion is in a semi-opened position in the leaflet resection group compared to the leaflet preservation group. We also evaluated how the restriction of mitral valve mobility affects postoperative mitral valve functions. The postoperative MVA or transmitral pressure gradient (TMPG) did not differ between the two groups.

Elevated postoperative TMPG, especially the presence of functional mitral stenosis (MS), is an important outcome to assess after MV repair because functional MS is associated with lower exercise capacity, heart failure and poor long-term prognosis [13, 14]. Annuloplasty using small-sized rings, especially those smaller than 28 mm, reportedly increased postoperative mean TMPG [15]. In addition, MVA is an independent predictor of exercise capacity, and MVA ≤ 1.5 cm^2^ is associated with higher resting mean TMPG [16].

We hypothesized that the restriction of mitral valve mobility caused by the leaflet resection techniques leads to ‘mitral stenosis-like’ morphological changes in the mitral valve, resulting to a decrease in MVA and an increase in TMPG. In this study, however, annuloplasty ring size, indexed ring size, postoperative MVA, and peak and mean TMPG did not differ significantly between the two groups. Our findings revealed that the mobility of the resected posterior mitral leaflet was mainly restricted to its closing motion during systole, and the opening motion during diastole was not restricted. This is the reason why there is no obvious difference in TMPG after MV repair between the leaflet resection and the leaflet preservation technique.

In this study, there is a bias in selecting the type of ring used for annuloplasty between the two groups. Therefore, it is necessary to consider the possibility that it affected TMPG. Chan et al. showed a good correlation between postoperative MVA and the size of the complete ring or partial ring [16]. In addition, they reported that MVA was greater in patients with partial rings than in those with complete rings [16]. However, in the present study, there was no difference not only in the ring size but also in postoperative MVA. Therefore, the impact of the selected type of annuloplasty ring on TMPG is considered minimal.

With regard to cardiac functions, postoperative LVEF tended to be lower in the leaflet resection group, despite no significant difference in preoperative LVEF between the two groups. LVEF declines immediately after MV surgery, and the decrease in LVEF is associated with preoperative left heart dilatation (left ventricular end-systolic diameter, left ventricular end-diastolic diameter, and left atrial size), left ventricular (LV) dysfunction, and the surgical procedures performed [17, 18]. Chronic left ventricular volume overload due to mitral regurgitation causes compensatory dilatation of the left ventricle [19]. In addition, left ventricular contractility is irreversibly impaired in some patients with mitral regurgitation [20]. In this study, preoperative LV end-systolic and end-diastolic volumes tended to be greater in the leaflet resection group. This suggested that patients in the leaflet resection group tended to undergo surgery at a later stage than those in the leaflet preservation group, and that irreversible left ventricular damage might have occurred preoperatively in the leaflet resection group. In addition, Imasaka et al. reported that LVEF was better preserved in patients who underwent MV repair with chordal replacement than in those who underwent leaflet resection 1 month after MV surgery because of superior preservation of mitral-ventricular continuity [18]. In this study, three patients in the leaflet resection group (among 14 patients) did not undergo artificial chordal implantation, which may have negatively affected LV function.

When the resected leaflet is reconstructed, other normal chordae tendineae shift their position toward the center of the posterior leaflet. The native chordae tendineae of the reconstructed posterior leaflets are relatively shortened from the papillary muscles to which they are attached. As a result, the reconstructed leaflet is pulled toward the left ventricular side, and the leaflet motion is thought to be restricted to the semi-opened position.

In the present study, there were no differences in mitral valve function between the two surgical techniques. However, the mobility of mitral valve leaflet was obviously restricted by the leaflet resection. Therefore, it is considered preferable to perform the leaflet preservation technique as much as possible in terms of preserving MV mobility.

## Limitations

This study has several limitations that should be considered when interpreting the results. First, this was a retrospective study with all of the inherent limitations. Secondly, this study included a relatively small number of patients who underwent MV repair during this period. The primary purpose of the present study was to evaluate the effects of leaflet resection on the MV mobility. Therefore, we excluded all segment lesions other than P2 prolapse to eliminate measurement errors in the echocardiography. In addition, we excluded patients who underwent aortic valve replacement during the same operation because of the impact of other valvular diseases on the left ventricle. Although this reduced the number of study patients, we believe it allowed us to accurately assess the MV mobility and the cardiac functions. Thirdly, two types of rings were used in this study. Both rings are semi-rigid ring. Regardless of which ring is chosen, the posterior leaflet mobility may be affected by the attachment of the ring in all cases. However, it is unclear whether different types of rings affect the leaflet mobility differently. Finally, we evaluated patients after MV repair in the early postoperative period. Therefore, further longitudinal follow-up studies are warranted to address limitations of the current study.

## Conclusions

The leaflet preservation technique preserves the mobility of the corrected leaflet more effectively compared to the leaflet resection technique in the MV repair. One of the general principles of successful mitral valve repair is that leaflet motion should be restored or preserved [21]. Therefore, the leaflet preservation technique is more preferable than the leaflet resection technique at preserving the corrected leaflet mobility.

## Data Availability

No datasets were generated or analysed during the current study.
